# Dynamic Vision-Based Non-Contact Rotating Machine Fault Diagnosis with EViT

**DOI:** 10.3390/s25175472

**Published:** 2025-09-03

**Authors:** Zhenning Jin, Cuiying Sun, Xiang Li

**Affiliations:** 1 Key Laboratory of Education Ministry for Modern Design and Rotor-Bearing System, Xi’an Jiaotong University, Xi’an 710049, China; 2 State Key Laboratory of Engine and Powertrain System, Weichai Power Co., Ltd., Weifang 261061, China; suncuiy@weichai.com

**Keywords:** eagle vision transformer, deep learning, dynamic vision, event-based camera, fault diagnosis

## Abstract

Event-based cameras, as a revolutionary class of dynamic vision sensors, offer transformative advantages for capturing transient mechanical phenomena through their asynchronous, per-pixel brightness change detection mechanism. These neuromorphic sensors excel in challenging industrial environments with their microsecond-level temporal resolution, ultra-low power requirements, and exceptional dynamic range that significantly outperform conventional imaging systems. In this way, the event-based camera provides a promising tool for machine vibration sensing and fault diagnosis. However, the dynamic vision data from the event-based cameras have a complex structure, which cannot be directly processed by the mainstream methods. This paper proposes a dynamic vision-based non-contact machine fault diagnosis method. The Eagle Vision Transformer (EViT) architecture is proposed, which incorporates biologically plausible computational mechanisms through its innovative Bi-Fovea Self-Attention and Bi-Fovea Feedforward Network designs. The proposed method introduces an original computational framework that effectively processes asynchronous event streams while preserving their inherent temporal precision and dynamic response characteristics. The proposed methodology demonstrates exceptional fault diagnosis performance across diverse operational scenarios through its unique combination of multi-scale spatiotemporal feature analysis, adaptive learning capabilities, and transparent decision pathways. The effectiveness of the proposed method is extensively validated by the practical condition monitoring data of rotating machines. By successfully bridging cutting-edge bio-inspired vision processing with practical industrial monitoring requirements, this work creates a new paradigm for dynamic vision-based non-contact machinery fault diagnosis that addresses critical limitations of conventional approaches. The proposed method provides new insights for predictive maintenance applications in smart manufacturing environments.

## 1. Introduction

Over the past few decades, intelligent machinery fault diagnosis methods have garnered considerable attention. Driven by the swift progress in artificial intelligence technologies, data-intensive fault diagnosis approaches have made remarkable achievements in machine maintenance [1,2,3]. Current studies employ diverse signal modalities for machinery health assessment [4], such as vibrational, electrical, acoustic, and emission-based measurements. Notably, vibration analysis has proven especially effective in characterizing equipment condition, given that mechanical faults typically induce measurable oscillatory responses during operation. Accelerometers [5] have become a common choice for vibration signal acquisition, and the majority of studies on machine fault diagnosis rely on vibration data obtained from accelerometers. Nevertheless, contact sensors are not always the preferred option for machine condition monitoring in numerous industrial settings [6]. In contrast, non-contact sensing eliminates the need for physical attachment, drastically reducing installation time, labor cost, and the risk of sensor-induced mechanical imbalance or resonance. It averts contamination and wear in corrosive, high-temperature, or high-speed environments, ensuring continuous operation without periodic sensor replacement or recalibration. Because there is no mechanical coupling, the technique measures the true surface motion of the target, free from mass-loading effects or frequency distortion. This preserves the fidelity of high-frequency and low-amplitude vibration signatures that are critical for early fault detection. Additionally, non-contact devices such as event-based or laser sensors can monitor multiple machines from a safe standoff distance, simplifying retrofitting on legacy equipment and enabling unobtrusive surveillance of sealed or rotating assemblies where cabling is impossible.

Convolutional Neural Networks (CNNs) [7] have achieved remarkable success in the field of computer vision, driven by advancements in deep learning technologies and hardware computing capabilities. Their effectiveness can be largely attributed to the pyramidal structure of CNNs and their inherent inductive biases, such as translation invariance and local sensitivity. Despite their success in computer vision tasks, Convolutional Neural Networks face several limitations when applied to mechanical fault diagnosis [8]. These limitations include inadequate modeling of global contextual information, computational complexity, sensitivity to data quality and noise, lack of sensitivity to local features and details, and data-intensive training requirements.

**Figure 1:**
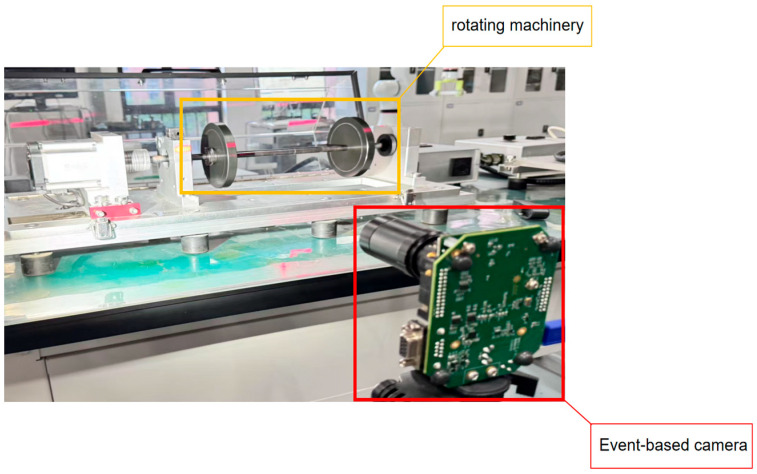
Schematic diagram of the dynamic vision data collected by the event-based camera.

This article presents a novel signal modality, dynamic machine vision data [9,10], and utilizes Eagle Vision Transformers (EViTs) [11,12,13] model to process the event data, thereby achieving minimally contact vibration measurement and accurate fault diagnosis of machinery. Dynamic vision data are captured by event-based cameras [12,13], as shown in Figure 1, which emulate the functionality of biological retinas and have been commercially available over the past decade.

These cameras are asynchronous sensors that record per-pixel brightness changes, referred to as events, instead of capturing the brightness values of all pixels within a frame. In contrast to traditional standard cameras, event-based cameras offer substantial advantages for industrial applications, such as high temporal resolution, high dynamic range, high-speed motion estimation, low latency, and low power consumption. Event-based cameras are particularly effective for addressing dynamic scene sensing challenges, including motion recognition, high-speed counting, and drone vision. Specifically, their microsecond-level temporal resolution (up to 10 kHz equivalent frame rate) enables the capture of impulsive vibrations or tool chatter that would be smeared out by conventional 30–120 fps imagers, while a dynamic range exceeding 120 dB allows for reliable operation under the extreme lighting contrasts found near welding arcs or bright conveyor belts. Because each pixel operates autonomously, data throughput—and thus latency—scales only with scene dynamics; in practice, end-to-end delays below 1 ms are routinely achieved on edge hardware, and idle pixels consume mere microwatts, yielding a 10–100× power reduction versus streaming RGB sensors. These merits translate directly to industrial use cases: on high-speed spindles, event cameras track sub-micron run-out by observing microscopic laser-dot displacements without additional encoders; on bottling lines, they count up to 50,000 parts per second by detecting the edges of passing caps with zero motion blur; in steel mills, they monitor strip-flatness in real time by measuring the vibration of reflective edges under strong ambient glare; and on autonomous forklifts, they provide robust obstacle detection when sudden brightness changes (e.g., exiting a dark aisle into sunlight) would saturate conventional vision.

EViTs integrate the unique physiological and visual characteristics of eagle eyes with the architecture of vision transformers, thereby harnessing the potential advantages of both. Unlike CNNs, which rely on fixed local kernels and full-frame grids, EViT replaces convolution with global Bi-Fovea Self-Attention that operates directly on sparse, timestamped events. The Bi-Fovea Visual Interaction (BFVI) structure of EViTs is designed to combine the benefits of both cascaded and parallel architectures, including hierarchical organization and parallel information processing. Building on this foundation, EViTs employ a novel Bi-Fovea Self-Attention (BFSA) mechanism [13] and a Bi-Fovea Feedforward Network (BFFN). These components mimic the hierarchical and parallel information processing scheme of the biological visual cortex. As improved variants of self-attention and feedforward networks, respectively, BFSA and BFFN enable the network to extract features in a coarse-to-fine manner, resulting in high computational efficiency and scalability. Its coarse-to-fine BFVI pipeline cuts computation while preserving microsecond timing, enabling low-power, minimally intrusive monitoring of industrial machinery.

Although event-based sensing holds substantial promise for machine fault detection, its practical implementation faces notable technical hurdles. The operational paradigm of event cameras differs fundamentally from traditional imaging systems, demanding specialized computational frameworks for reliable anomaly detection. To our knowledge, this work pioneers the systematic investigation of this research gap. The key innovations of this article are listed as follows.

1. A novel non-contact fault diagnosis method based on dynamic vision sensing is proposed. Experimental results demonstrate the viability of utilizing dynamic vision data acquired from event-based cameras for mechanical fault detection.
2. The EViT model is proposed for the first time to process vision data, addressing a critical research gap in mechanical fault diagnosis applications.
3. Experimental validation was conducted using real-world rotor machinery data to verify the performance of the EViT model for mechanical fault diagnosis.

The rest of this article is organized as follows. It starts with the related works in Section 2. The proposed method is presented in Section 3, and experimentally validated in Section 4. Finally, Section 5 concludes this article.

## 2. Related Work

### 2.1. Intelligent Machinery Fault Diagnosis

Industrial equipment reliability critically depends on effective condition monitoring systems. Precise malfunction identification at early stages significantly improves operational security while optimizing upkeep expenditures [14,15,16]. Recent advances in computational intelligence [17] have led to substantial progress in automated equipment failure detection techniques. Among various sensing modalities, oscillatory motion measurements remain the predominant data source for equipment wellness assessment, with vibration analysis consistently demonstrating superior diagnostic performance. Innovative signal processing techniques, such as the enhanced empirical mode decomposition approach developed by Wang [16] and colleagues, have shown remarkable efficacy in isolating critical failure signatures from mechanical vibration patterns. Complementary research by Martin-Diaz [18] and collaborators established a pioneering technique for incipient defect identification in electric motors through sophisticated analysis of electromagnetic signatures across temporal and spectral domains, incorporating adaptive boosting with refined data acquisition strategies. Alternative monitoring approaches, including stress wave detection methodologies, have also proven valuable, as evidenced by successful applications in rail transportation bearing systems [19].

The advent of sophisticated connectionist systems has revolutionized equipment failure analysis, with hierarchical learning architectures demonstrating exceptional proficiency in deciphering intricate data correlations [20,21,22]. Modern diagnostic systems increasingly incorporate diverse neural network topologies, including spatial feature extractors, temporal sequence analyzers, and memory-enhanced architectures, to process mechanical oscillation data. Notable contributions include Shao’s hierarchical feature learning system for rotational equipment [23], employing deep autoencoding structures for automated signature extraction and classification. Jia’s team [24] advanced the field through their normalized spatial filtering network, specifically designed for handling uneven failure category distributions, with incorporated feature visualization for model interpretability. Yu’s innovative one-dimensional convolutional architecture demonstrated superior performance in vibration-based failure categorization [5], incorporating bilateral weighting mechanisms for enhanced generalization to novel fault conditions. Parallel developments by Huang [25] introduced sophisticated learning systems for fluid power apparatus diagnostics, enabling automated processing of heterogeneous temporal data without requiring specialized domain knowledge.

Contemporary research predominantly focuses on acceleration-based monitoring systems, with relatively limited exploration of alternative sensing modalities. Particularly scarce are investigations into non-contact optical measurement techniques for mechanical vibration analysis, representing a significant gap in current condition monitoring literature.

### 2.2. Event-Based Machine Vision

Bioinspired vision sensors, emerging over the past twenty years, have revolutionized dynamic scene capture through their unique ability to record asynchronous pixel-level luminance variations which are termed events [26]. These neuromorphic imaging devices and their generated data streams possess distinct advantages including minimal energy requirements and exceptional temporal precision, making them particularly suitable for applications demanding ultra-fast motion analysis. Contemporary research has demonstrated the versatility of these sensors across diverse fields, ranging from 3D scene reconstruction and motion vector calculation to robotic navigation, image enhancement, and microscopic particle tracking [27].

The automotive industry has particularly benefited from these innovative sensors, with numerous driver assistance systems now incorporating event-based visual processing [15]. The sensors’ wide dynamic ranges and near-instantaneous responses enable superior vehicular perception capabilities. Notable implementations include Zhou’s navigation system [28] that maintains reliable operation under extreme lighting variations while requiring only standard computational resources. Jin’s team [29] developed a sophisticated six-degree-of-freedom position estimation framework combining convolutional and recurrent neural architectures for processing event-based visual streams, achieving both computational efficiency and precision. Another breakthrough came from Lagorce’s motion tracking algorithm [30], which exploits spatiotemporal event correlations to enhance system resilience while effectively solving object recognition challenges through innovative optical flow computation in velocity-direction coordinates.

Despite these advancements, the potential of dynamic vision in mechanical system monitoring remains largely unexplored. Traditional visual inspection techniques face inherent limitations in vibration analysis due to noise susceptibility and motion capture constraints. This investigation systematically evaluates the applicability of neuromorphic sensing for equipment diagnostics, presenting comprehensive experimental validation of its effectiveness for fault detection applications.

## 3. Event-Based Fault Diagnosis Method

### 3.1. Event Vision Data and Representations

This research presents a novel framework for equipment fault detection utilizing dynamic vision technology. The event-based camera generates asynchronous data streams where individual events encode discrete brightness variations at specific pixels and timestamps. Each event comprises a four-dimensional vector e = [x, y, t, p], with (x,y) indicating spatial coordinates, t representing the precise timing, and p ∈ {−1, +1} denoting brightness decrease or increase, respectively. These sensors operate by continuously monitoring pixel-level intensity changes and triggering events only when variations exceed predetermined thresholds.

**Figure 2:**
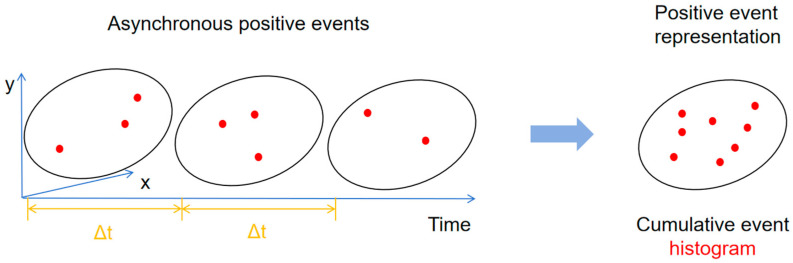
Illustration of the generation of the event representations. The positive event representation is shown for instance.

For mechanical system monitoring applications, the event vision data in the time sequence are recorded as $\{e_{i}{\}}_{i=1}^{n_{e}}$, where $e_{i}$ denotes the ith event, and $n_{e}$ is the number of all the events in the concerned data collection time period $t_{e}$. The inherent asynchronicity and sparse nature of event data pose unique challenges for conventional deep learning approaches designed for regular 1D temporal or 2D spatial data. To address this, we introduce a novel two-channel image-like representation that preserves the spatiotemporal characteristics of event streams. As depicted in Figure 2, this representation separately accumulates positive and negative polarity events at each pixel location, creating complementary information channels that capture the dynamic evolution of machine vibrations.

The proposed methodology establishes a formal training framework ${𝒟}_{train}={\left\{\left(r_{i},h_{i}\right)\right\}}_{i=1}^{n_{train}}$, where each sample $r_{i}=[r_{i}^{+},r_{i}^{-}]$ consists of polarity-separated event count matrices with dimensions Nx × Ny matching the sensor resolution. And $h_{i}$ means the corresponding machine health condition label, $n_{train}$ is the number of the training samples, while $r_{i}^{+}$ and $r_{i}^{-}$ are the channels for the positive and negative cumulative event number, respectively. The corresponding health state labels $h_{i}$ enable supervised learning of the diagnostic model. All training samples maintain consistent event counts, which are denoted as $n_{event}$, to ensure dimensional uniformity. The core technical objective involves developing a deep neural network (EViTs) mapping function f that establishes the relationship h = f(r) between event-based representations and equipment health conditions.

### 3.2. Deep Neural Network Model

**Figure 3:**
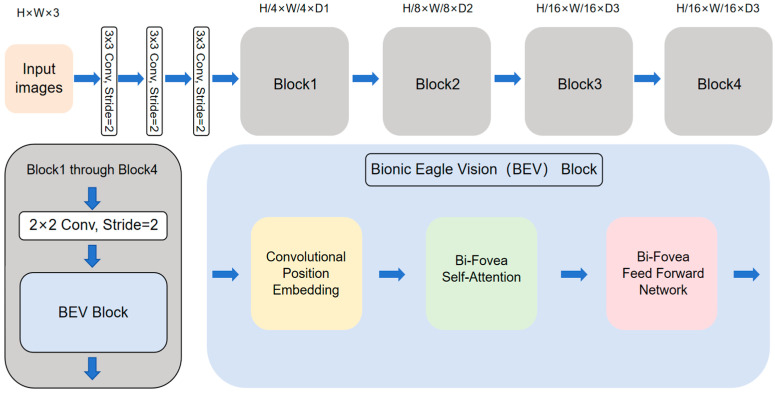
Illustration of the EViT. The EViT architecture features a convolutional stem followed by a four-stage pyramid. Within each stage, a 2 × 2 convolution operating at a stride of 2 precedes multiple Bionic Eagle Vision (BEV) blocks. Structurally, every BEV block integrates three core elements: a Convolutional Positional Embedding (CPE), a Bi-Fovea Self-Attention (BFSA) module, and a Bi-Fovea Feedforward Network (BFFN).

Our research concentrates on improving both event data interpretation and diagnostic reliability for mechanical fault detection. The central innovation involves implementing a specialized deep neural network, which is Eagle Vision Transformers (EViTs) [13], whose architecture is visually documented in Figure 3.

The architectural framework of EViT incorporates three fundamental components: a convolutional stem, multiple 2 × 2 convolutional operations, and Bionic Eagle Vision (BEV) modules. Critical implementation details include: the 2 × 2 convolutional operators employ a stride setting of 2 layers to facilitate patch embedding; adhering to established hierarchical paradigms [31,32,33], the network organizes its computation into four structurally homologous stages; and progressive feature resolution reduction (4×, 8×, 16×, and 32× scaling factors across stages 1–4, respectively) coupled with channel dimension expansion (D1 through D4).

Processing flow initiates with an H × W × 3 dimensional input undergoing initial feature extraction via the convolutional stem—a triple-stacked 3 × 3 convolutional configuration where the initial layer’s stride-2 operation enhances early-stage training stability. Subsequent feature transformation occurs through an alternating sequence of 2 × 2 convolutional layers and BEV modules, progressively building multi-scale target representations. For mechanical fault classification applications, the system terminates with a classification head comprising layer normalization, global average pooling, and a final fully connected projection layer to generate diagnostic predictions.

As the foundational structural elements of EViTs, BEV blocks synergistically combine the strengths of convolutional operations and visual transformer architectures. Each BEV block incorporates three core modules: a Convolutional Position Embedding (CPE) mechanism, a Bi-Fovea Self-Attention (BFSA) module, and a Bi-Fovea Feedforward Network (BFFN). Spatial relationships are fundamentally important for characterizing visual data representations. Conventional vision transformer implementations typically employ two approaches for position encoding: Absolute Position Embedding (APE) [34] and Relative Position Embedding (RPE) [35]. These embedding schemes generally utilize either sinusoidal functions with different frequency parameters or trainable parameter matrices.

APE implementations face notable limitations as they are resolution-dependent, requiring modification when feature token dimensions change, due to their lack of scale adaptability. Conversely, RPE mechanisms account for inter-token spatial relationships within input sequences, demonstrating translation-invariant characteristics. Nevertheless, RPE introduces computational overhead when determining pairwise feature token distances. More critically, computer vision applications often demand absolute positional data, which RPE cannot inherently supply.

To address these challenges, EViT first embeds every token with Convolutional Position Embedding (CPE). By replacing sinusoidal or learned absolute/relative encodings with a light-weight depth-wise convolution, CPE gains two unique properties: (i) zero-padded convolutions let the same layer adapt to arbitrary input resolutions without re-training, yielding true plug-and-play behaviour, and (ii) the inductive locality inherent in depth-wise kernels injects translation-equivariance into the otherwise bias-free Transformer, lifting the model’s effective capacity ceiling.

Within each Bionic Eagle Vision (BEV) block, CPE’s positional features are processed by the Bi-Fovea Self-Attention (BFSA) mechanism. Mimicking the eagle’s shallow and deep foveae, BFSA splits computation into a coarse global branch (Shallow-Fovea Attention) that downsamples keys/values to capture scene gist, and a fine-grained branch (Deep-Fovea Attention) that re-uses the global summary to refine every spatial location. Their outputs are additively fused, achieving simultaneous wide-field context and pinpoint detail without cascading stages or heavy dense maps.

Complementing BFSA, the Bi-Fovea Feed-Forward Network (BFFN) adopts a two-scale depth-wise design: an initial 3 × 3 kernel enlarges receptive fields to harvest local textures, followed by a 1 × 1 projection that mixes channels and re-weights features. GELU non-linearity and residual paths are retained to stabilize gradients. Together, CPE, BFSA and BFFN form a unified “coarse-to-fine yet parallel” pipeline that marries the efficiency of CNNs with the expressiveness of self-attention, all within a single, scalable block.

### 3.3. Loss Function Method

This article introduces an advanced loss function method to optimize the discriminative capability for mechanical fault pattern recognition. CrossEntropyLoss [36], also known as categorical cross-entropy, is a widely used loss function in deep learning for classification tasks. It measures the dissimilarity between the predicted probability distribution and the true label distribution, guiding the model to adjust its parameters to minimize this discrepancy. Unlike regression losses such as Mean Squared Error (MSE), CrossEntropyLoss is specifically designed for probabilistic classification, making it more effective for tasks where outputs represent class probabilities.

Given a true label y (typically one-hot encoded) and a predicted probability p (obtained via Softmax activation), the CrossEntropyLoss for a single sample is defined as:

(1) $$L_{CE}=-\sum_{i=1}^{C}y_{i}\log(p_{i})$$

(2) $$p_{i}=\frac{e^{z_{i}}}{\sum_{j=1}^{C}e^{z_{j}}}$$

where C is the number of classes. Since $y_{i}$ is non-zero only for the correct class, the loss simplifies to the negative log-likelihood of the true class probability. This formulation penalizes incorrect predictions more severely as p approaches zero, ensuring rapid gradient updates during training. Before computing CrossEntropyLoss, raw model outputs(logits) [37] are transformed into probabilities using the Softmax function. Softmax normalizes logits into a probability distribution, ensuring $\sum_{i=1}^{C}p_{i}=1$. This step is crucial because CrossEntropyLoss operates on probabilities rather than unbounded logits.

### 3.4. General Implementation

**Figure 4:**
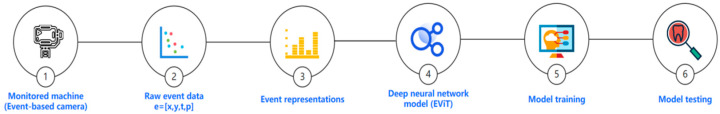
General implementation flowchart of the proposed event vision- based fault diagnosis method.

Figure 4 presents the operational pipeline of our novel event-vision based approach for intelligent mechanical fault detection [38,39,40,41]. The system workflow initiates with data acquisition, where event-based cameras capture dynamic visual streams from operational machinery across various health states to create annotated training datasets. These asynchronous event streams undergo specialized preprocessing to generate structured event representations suitable for computational analysis.

The training phase commences with initialization of the deep neural network model (EViTs), which subsequently undergoes iterative optimization using the annotated dataset [42,43,44,45]. During each epoch, the algorithm first selects a random minibatch from the original samples and generates complementary augmented instances.

The training procedure follows an iterative optimization cycle where samples are dynamically generated and utilized within each epoch. Upon completing parameter updates for a given epoch, the temporarily generated augmented instances are purged from memory before initiating the subsequent training cycle [46,47,48]. This process involves: reapplication of the data augmentation protocol to create fresh synthetic samples, forward-backward propagation through the network architecture, and continuous repetition of this sequence until convergence criteria are satisfied. The evaluation phase subsequently assesses model generalization capability by processing previously unseen unlabeled test data through the trained network to quantify diagnostic accuracy.

## 4. Experiments

### 4.1. Event Vision Dataset for Fault Diagnosis

**Figure 5:**
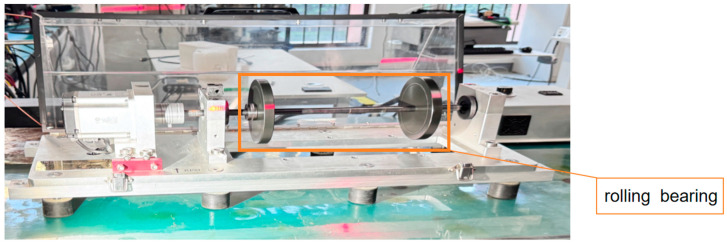
Test rig of the rotating machine condition monitoring problem in this study.

**Figure 6:**
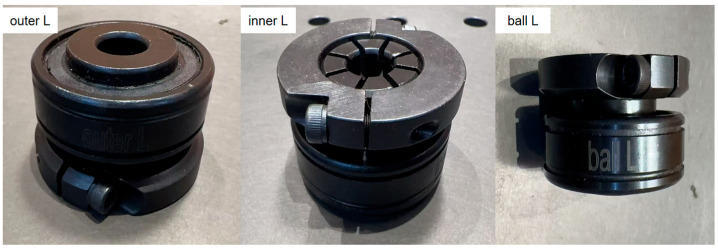
Schematic diagrams of the three types of bearings.

This research evaluates the proposed methodology using experimental data acquired from a dedicated rolling element bearing test platform. As shown in Figure 5, the experimental setup comprises an electric motor driving a shaft supported by ER-16K bearings, designed to simulate four distinct mechanical health states: healthy operation, inner race defect, rolling element defect, and outer race defect, collectively establishing a multi-category fault identification challenge. The schematic diagrams of the three types of bearings used in the experiment are shown in Figure 6. Artificially induced defects with approximate 1mm depth were carefully introduced at various bearing locations using precision machining tools to replicate realistic failure modes. Comprehensive testing was conducted across three operational speeds (1200, 1500, and 1800 RPM), generating vibration datasets encompassing all fault conditions under varying rotational velocities. Vibration monitoring was performed using a strategically positioned accelerometer mounted directly on the bearing housing, with all measurements captured at 12.8 kHz sampling frequency to ensure adequate temporal resolution for fault signature analysis.

A third-generation Prophesee neuromorphic vision sensor (Gen 3.1) was positioned adjacent to the test bearing to acquire asynchronous event streams. The device specifications include a 640 × 480 pixel array, 200 μs event latency, and peak event throughput of 50 million events per second. Synchronized acquisition of vibration signals and event-based visual data was performed across multiple rotational velocities and bearing conditions under controlled illumination. For targeted vibration analysis, a 64 × 64 pixel region centered on the bearing housing was isolated from the raw event data for subsequent fault detection processing.

### 4.2. Fault Diagnosis Tasks and Comparisons

The dynamic vision data is evaluated for fault diagnosis in this study. For the event vision data, 2000 events are considered in each sample, which is prepared using the method described in Section 3.1. The experimental configuration employs a fixed sample length of 4096 data points, with each fault diagnosis task (combining specific health states and rotational speeds) containing 500 training instances and 250 testing instances. To comprehensively assess diagnostic performance of different deep neural network models, we conduct parallel evaluations across four distinct fault detection scenarios using both CNN and EViTs models. The comprehensive information of the related fault diagnosis tasks is presented in Table 1.

This study conducted comparative analyses of fault diagnosis performance using diverse deep neural network architectures. For tasks A1 through A4, conventional CNN models were employed to process event-based vision data, while tasks B1 to B4 utilized the proposed EViT framework as detailed in Section 3.2 for event data analysis. The experimental configurations and data acquisition procedures remain basically consistent with the proposed method. Classification accuracy serves as a well-established evaluation criterion in pattern recognition applications, particularly suitable for mechanical fault detection scenarios. This metric is consequently adopted for performance assessment throughout our experimental analysis. Formally, given a test set containing $n_{test}$ samples, where $n_{correct}$ represents the count of accurately classified instances matching the true fault labels, the diagnostic accuracy is computed as ($n_{correct}$/$n_{test}$) × 100%. The complete parameter configuration is documented in Table 2, with parameter optimization playing a critical role in our methodology.

As delineated in Table 2, the parameters are systematically categorized into: (1) trainable network parameters θ, and (2) tunable hyper-parameters. Regarding the model parameters, they are optimized within the deep neural network framework using training data and preset hyper-parameters. These parameters can be determined once the hyper-parameters and training data are provided, although minor variations may occur across different training runs due to inherent model stochasticity. For this study, both the CNN and EViT models were configured with identical fundamental parameters. Hyper-parameter optimization follows standard data-driven machine learning practices through dedicated validation procedures. In our experimental framework, this involves: (1) establishing a validation set under 1200 r/min operating conditions with 1000 training and 1000 test samples, which is completely independent from tasks A1–A4 datasets, and (2) adopting 300 training epochs as the benchmark based on observed convergence behavior and evidenced by stabilized loss metrics. For real-world deployment, the parameter configuration process maintains methodological consistency: operational data from target equipment forms the validation basis for hyper-parameter tuning. While field applications often face data scarcity challenges, two practical solutions are implemented: minimum viable validation using binary health states (normal/faulty), or temporal segmentation of extended unlabeled operational records into distinct condition periods. This approach ensures rigorous parameter optimization while accommodating industrial implementation constraints.

### 4.3. Experimental Results and Performance Evaluations

Table 3 presents the comprehensive experimental results of different deep learning models across various fault diagnosis tasks. The proposed model consistently achieves testing accuracies above 95% under different operating conditions, demonstrating significant advantages for mechanical fault diagnosis problems and thereby validating the effectiveness of the event-vision-based diagnostic paradigm. All evaluated tasks exhibit similar patterns of result distribution.

Furthermore, this study conducts a comparative analysis of diagnostic performance between CNN and EViT models. Taking task B1 as an example, the EViT model achieves a testing accuracy of 99.0%, slightly outperforming the CNN model’s 96.1% accuracy, with similar performance trends observed across other tasks. These results demonstrate the superior competitiveness of EViT models over conventional CNN architectures for mechanical fault diagnosis applications. The experiments were implemented on a hardware platform consisting of a GeForce RTX 4060ti GPU and Intel i7 CPU, utilizing the PyTorch 1.12 programming framework. Computational efficiency tests reveal average training times of 672.5 s for tasks A1–A3 and 2136.7 s for task A4, indicating computationally acceptable overhead for an offline diagnostic approach.

**Figure 7:**
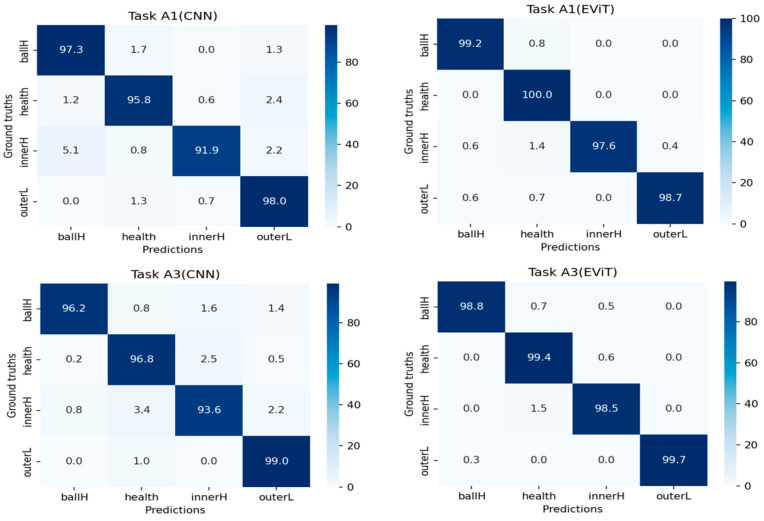
Confusion matrices of two models in the fault diagnosis tasks A1 and A3.

The comparative performance evaluation of different diagnostic models is illustrated in Figure 7, which presents the confusion matrices for both CNN and EViT architectures when processing tasks A1 and A3. The experimental data reveals remarkable diagnostic capabilities across all fault categories, with classification accuracy consistently surpassing 95% for each fault mode while maintaining minimal false positive rates—findings that align perfectly with the outstanding test accuracy documented in Table 3. Notably, the transformer-based EViT approach demonstrates significantly better fault identification performance than conventional CNN, particularly in detecting mechanical anomalies, thereby conclusively confirming the methodological advantages of the proposed framework.

In practical mechanical systems, operational conditions often exhibit significant variability. This study evaluates the performance of CNN and EViT models under such condition shifts, utilizing fault diagnosis datasets with wide operational ranges due to limited availability of specialized rotating machinery event datasets. As detailed in Table 4, eight cross-condition tasks were designed: Tasks C1 and C2 incorporate both 1200 rpm (limited) and 1500 rpm (abundant) training data but test on 1200 rpm, whereas Tasks C3 and C4 exclude 1500 rpm data entirely. Comparative analysis of C1 versus C3 and C2 versus C4 reveals that models trained only on limited same-condition data (C3 and C4) achieve suboptimal accuracy, while introducing cross-condition data (C1 and C2) yields substantial improvements—particularly for EViT, which shows greater performance gains than CNN when leveraging heterogeneous operational data (C2 and D2 compared to C1 and D1).

These results demonstrate that cross-condition training data effectively compensates for single-condition data scarcity, enhancing model robustness to operational variations. EViT’s superior adaptability stems from its inherent capacity to extract condition-invariant features: its attention mechanism aligns naturally with the sparse spatiotemporal characteristics of mechanical event data, while its explicit clustering of feature distributions across conditions contrasts with CNN’s implicit learning approach that remains more sensitive to domain shifts. This architectural advantage allows EViT to more fully exploit complementary information embedded in multi-condition datasets, establishing it as a more versatile solution for real-world applications where operational parameters fluctuate.

**Figure 8:**
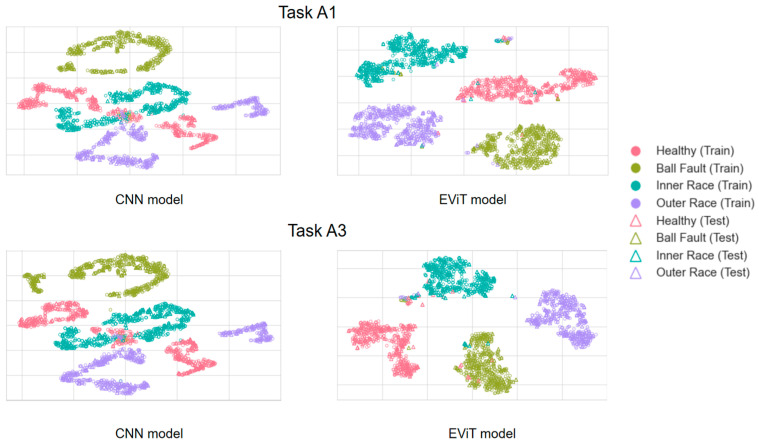
Visualization results of the learned features from the data by different models in the tasks A1 and A3.

Subsequently, visual validation was performed on the features extracted from event data using different methods. Specifically, the fully connected features from the final layer of the deep neural network were extracted and visualized through t-SNE dimensionality reduction. The results for tasks A1 and A3 are presented in Figure 8. The visualization demonstrates that compared to the CNN model, samples processed by the EViT model exhibit tighter intra-class clustering and more distinct inter-class boundaries across different health states. This clearly illustrates EViT’s superior effectiveness in event data-based fault pattern recognition.

To comprehensively validate the performance advantages of EViT over conventional CNNs, Table 5 presents a comparative analysis of diagnostic accuracy among different models. As shown in Table 5, Task E1 employs the EViT model, while Tasks E2 and E3 utilize CNN architectures with varying network depths, and Task E4 adopts a CBAM-enhanced CNN model (CBAM-CNN), with all other experimental conditions maintained identical. The experimental results demonstrate that Task E1 achieves marginally higher accuracy compared to other tasks, thereby substantiating the superior diagnostic performance of the proposed EViT framework for mechanical fault detection.

**Figure 9:**
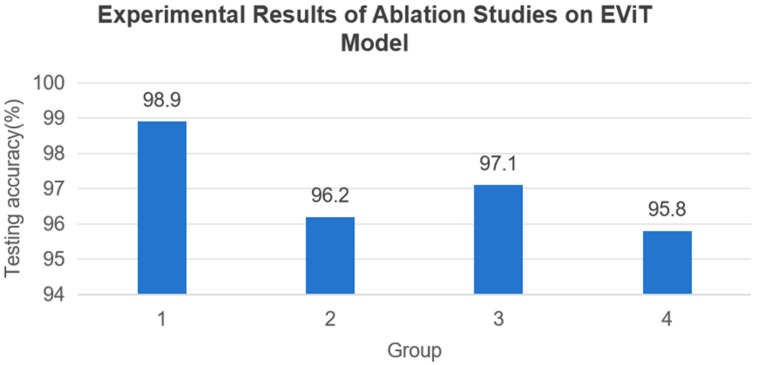
Effects of each module in the EViT model on fault diagnosis.

In order to validate the effectiveness of individual modules in the EViT model for fault diagnosis, we conducted ablation studies on the BFSA, BFFN, and CPE modules. Experimental Group 1 employed the complete EViT model, Group 2 replaced the BFSA with standard MHSA, Group 3 substituted the BFFN with a standard FFN, and Group 4 removed the Convolutional Position Embedding (CPE). All groups were tested on the identical Task A1 dataset. As shown in Figure 9, the experimental results demonstrate significant performance impacts when removing these modules. Specifically, replacing BFSA with standard MHSA caused a 2.5% accuracy decrease, while substituting BFFN with FFN reduced accuracy by 1.8%. The complete removal of CPE led to the most substantial performance degradation. These findings clearly establish the critical importance of all three modules for optimal model performance.

Moreover, conventional vibration-based fault diagnosis techniques predominantly rely on single-point data acquisition. However, adopting a machine dynamic vision approach enables monitoring of extended spatial regions beyond the immediate target component. Notably, bearing defects can generate minute disturbances in shaft dynamics that often elude detection by conventional sensors. This research systematically examines how varying event frame regions of interest (ROI) affect model performance, evaluating both CNN and EViT architectures across multiple ROI configurations: the default 200 × 300 pixel area plus three bearing-centered regions measuring 100 × 200, 250 × 350, and 300 × 400 pixels.

**Figure 10:**
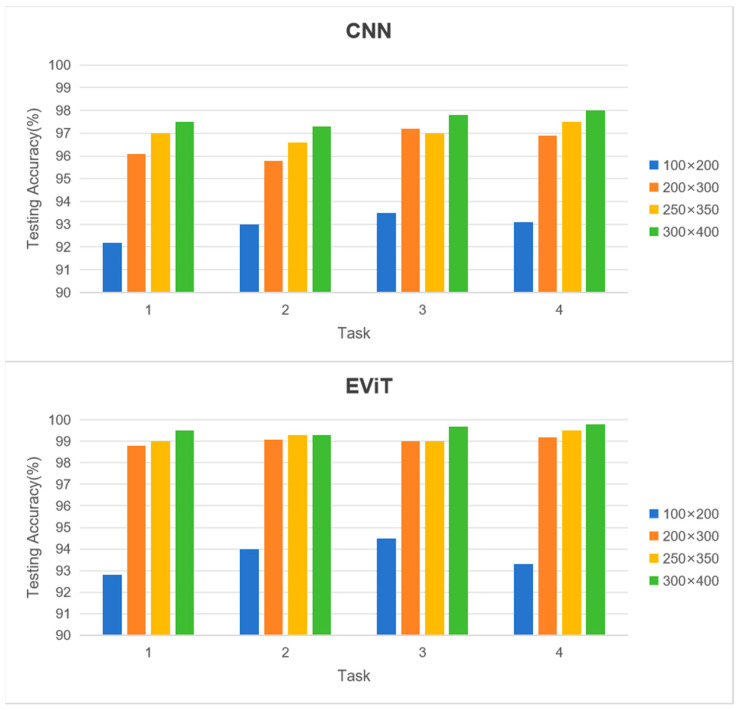
Effects of the ROI of the event frame on the fault diagnosis model performance in different tasks.

As evidenced in Figure 10, experimental findings reveal a general trend where expanded ROIs correlate with enhanced classification accuracy for both architectures. This improvement stems from the incorporation of more comprehensive vibration signatures encompassing both bearing and shaft dynamics. Nevertheless, the performance gains diminish considerably when exceeding the 200 × 300 pixel threshold. Given the substantial computational overhead associated with larger sample dimensions, the 200 × 300 pixel ROI remains the optimal selection for practical implementation.

A particularly noteworthy observation concerns the differential impact of ROI scaling—the EViT architecture demonstrates significantly greater accuracy enhancement compared to its CNN counterpart as ROI dimensions increase. This phenomenon underscores EViT’s superior capacity for processing expanded input domains, attributed to its enhanced ability to capture extensive spatial dependencies and integrate global contextual features. Such characteristics substantiate the transformer-based model’s advantages in machine condition monitoring applications.

It should be noted that although the EViT model demonstrates superior performance over CNN in fault diagnosis tasks, both architectures face common challenges in processing event-based vision data. Firstly, vibration feature extraction through visual perception is inherently challenging, particularly in identifying subtle differences between various fault modes, which is constrained by the spatiotemporal resolution of event sensors and environmental noise interference. Secondly, current research has yet to establish standardized frameworks for feature extraction and fault recognition specifically for event vision data, leaving room for optimization in processing efficiency—both in CNN’s local convolutional characteristics and EViT’s global attention mechanism when handling event streams. As the first study to apply EViT to mechanical fault diagnosis, this work provides a benchmark investigation by systematically comparing the performance differences between these two architectures. The results demonstrate EViT’s advantages in both accuracy and cross-condition adaptability, promising for further investigation in this direction.

## 5. Conclusions

This article presents the first integration of event-based cameras with an Eagle Vision Transformer (EViT) to propose a novel non-contact fault diagnosis method for rotating machinery. As an innovative neural network architecture, the EViT model demonstrates the capability to distinguish features corresponding to different health states, thereby addressing a critical research gap in the application of EViT for mechanical fault diagnosis. Through a systematic comparison of EViT and conventional Convolutional Neural Networks (CNN) in processing event-based vision data, this work elucidates the performance differences between these two models in fault diagnosis tasks.

The research highlights three key advantages of EViT over CNN. First, the Bi-Fovea Self-Attention (BFSA) mechanism in EViT mimics the central-peripheral visual processing of eagle eyes, enabling multi-scale vibration feature extraction and overcoming the limitations of CNN’s localized receptive fields. Experimental results confirm that this mechanism more effectively identifies invariant features across varying rotational speeds, whereas CNN struggles to adapt due to the fixed kernel sizes of its convolutional layers. Second, EViT’s hierarchical feature fusion architecture, facilitated by the Bi-Fovea Feedforward Network (BFFN), achieves superior synergy between global and local features, outperforming CNN in diagnosing compound bearing faults. Third, EViT exhibits significantly enhanced spatiotemporal modeling capabilities for event data, particularly in large Region-of-Interest (ROI) scenarios like exceeding 200 × 300 pixels, where its accuracy improvement surpasses that of CNN. This advantage stems from the self-attention mechanism’s ability to capture long-range spatial dependencies.

Notably, EViT’s superiority is most pronounced in cross-condition tasks. When trained on multi-speed operational data, EViT demonstrates a greater improvement in generalization performance compared to CNN, owing to its explicit feature distribution alignment strategy. In contrast, CNN relies on implicit regularization techniques such as data augmentation, leading to higher performance variability under unseen operating conditions. These findings provide a new solution for variable-condition diagnosis in industrial applications.

However, the study also identifies two limitations of EViT. First, the quadratic spatial complexity of the Bi-Fovea Self-Attention mechanism increases FLOPs by four to seven times and GPU memory usage by three to five times, rendering real-time deployment impractical on resource-constrained edge devices. Second, the Transformer backbone requires substantial labeled data to achieve optimal performance; in small-sample scenarios, EViT’s accuracy drops below that of lightweight CNNs, nullifying its theoretical advantages. These insights highlight critical directions for future research, which will focus on enhancing the model’s environmental generalization capability and optimizing its network architecture.

## Figures and Tables

**Table 1:** Fault diagnosis tasks in this study.

Task	A1	A2	A3	A4
model	CNN	CNN	CNN	CNN
condition (r/min)	1200	1500	1800	1200, 1500, 1800
Training samples	2000	2000	2000	6000
Testing samples	1000	1000	1000	3000
Task	B1	B2	B3	B4
model	EViT	EViT	EViT	EViT
condition (r/min)	1200	1500	1800	1200, 1500, 1800
Training samples	2000	2000	2000	6000
Testing samples	1000	1000	1000	3000

**Table 2:** Parameters used in this study.

Parameter	Value	Parameter	Value
Batch size	16	$n_{event}$	1000
η	1 × 10^−4^	Optimizer	AdamW
Epochs	300	Activation Function	ReLU

**Table 3:** Testing accuracy of different methods in different fault diagnosis tasks.

Task	CNN	Task	EViT
A1	96.1 ± 0.2	B1	98.8 ± 0.3
A2	95.8 ± 0.3	B2	98.6 ± 0.2
A3	97.2 ± 0.3	B3	99.3 ± 0.2
A4	96.9 ± 0.2	B4	98.5 ± 0.2

**Table 4:** Experimental results in the fault diagnosis tasks with variations in machine operating conditions.

Task Name	Model Name	Training Sample No.	Training Condition (r/min)	Testing Sample No.	Testing Condition (r/min)	Testing Accuracy (%)
C1	CNN	200 2000	1200 1500	1000	1200	89.6
C2	EViT	200 2000	1200 1500	1000	1200	92.4
C3	CNN	200	1200	1000	1200	83.8
C4	EViT	200	1200	1000	1200	84.7
D1	CNN	200 2000	1500 1800	1000	1500	88.9
D2	EViT	200 2000	1500 1800	1000	1500	92.2
D3	CNN	200	1500	1000	1500	82.5
D4	EViT	200	1500	1000	1500	83.8

**Table 5:** Experimental results in the fault diagnosis tasks with different models.

Task	Model	Condition (r/min)	Testing Accuracy (%)
E1	EViT	1200	99.2
E2	Model 1	1200	96.7
E3	Model 2	1200	97.4
E4	Model 3	1200	98.6

## Data Availability

Dataset available on request from the author.
